# Response of the Cerebral Cortex to Resistance and Non-resistance Exercise Under Different Trajectories: A Functional Near-Infrared Spectroscopy Study

**DOI:** 10.3389/fnins.2021.685920

**Published:** 2021-10-13

**Authors:** Ping Shi, Anan Li, Hongliu Yu

**Affiliations:** ^1^Institute of Rehabilitation Engineering and Technology, University of Shanghai for Science and Technology, Shanghai, China; ^2^Shanghai Engineering Research Center of Assistive Devices, Shanghai, China

**Keywords:** functional near-infrared spectroscopy, motor cortex, upper limb movement, resistance movement, neurophysiology

## Abstract

**Background:** At present, the effects of upper limb movement are generally evaluated from the level of motor performance. The purpose of this study is to evaluate the response of the cerebral cortex to different upper limb movement patterns from the perspective of neurophysiology.

**Method:** Thirty healthy adults (12 females, 18 males, mean age 23.9 ± 0.9 years) took resistance and non-resistance exercises under four trajectories (T1: left and right straight-line movement; T2: front and back straight-line movement; T3: clockwise and anticlockwise drawing circle movement; and T4: clockwise and anticlockwise character ⁕ movement). Each movement included a set of periodic motions composed of a 30-s task and a 30-s rest. Functional near-infrared spectroscopy (fNIRS) was used to measure cerebral blood flow dynamics. Primary somatosensory cortex (S1), supplementary motor area (SMA), pre-motor area (PMA), primary motor cortex (M1), and dorsolateral prefrontal cortex (DLPFC) were chosen as regions of interests (ROIs). Activation maps and symmetric heat maps were applied to assess the response of the cerebral cortex to different motion patterns.

**Result:** The activation of the brain cortex was significantly increased during resistance movement for each participant. Specifically, S1, SMA, PMA, and M1 had higher participation during both non-resistance movement and resistance movement. Compared to non-resistance movement, the resistance movement caused an obvious response in the cerebral cortex. The task state and the resting state were distinguished more obviously in the resistance movement. Four trajectories can be distinguished under non-resistance movement.

**Conclusion:** This study confirmed that the response of the cerebral motor cortex to different motion patterns was different from that of the neurophysiological level. It may provide a reference for the evaluation of resistance training effects in the future.

## Introduction

Motor tasks involving upper limbs are very common, from simple daily life to complex high-tech tasks. It is essential to effectively evaluate the effects of upper limb movement. The evaluation of training effects of upper limb movement usually only considers the level of motor performance. In many cases, due to the participation of patients and physiological reasons, there will be different degrees of compensation. Therefore, it is necessary to evaluate whether the training achieves the expected effect from the neurophysiological level. Rehabilitation exercise therapy needs to be understood from the perspective of neuroscience, neurophysiology, and motor control (Lee et al., 2018).

It is reported that the attention of participants in the task is highly related to the complexity of the task, which directly affects the effect of exercise (Radovanovic et al., 2002). Ordinary mechanical single exercises cannot achieve the expected rehabilitation efficacy, and appropriate resistance movement can provide good functional and physiological benefits for various groups (Skarabot et al., 2020). However, some studies show that both human (Weier et al., 2012) and non-human primates (Glover and Baker, 2020) have changes in intracortical inhibitory interneurons during resistance movement. Although long-term resistance training can modify muscle morphology and improve muscle motor co-function, the increase of force production caused by short-term resistance training is mainly due to neural adaptation (Folland and Williams, 2007; Skarabot et al., 2020). But few studies have explored specific responses in the cerebral cortex during resistance movements. Moreover, exercise is not controlled by separate regions of the muscle or cerebral cortex but should be studied from a synergistic perspective.

Mohseni et al. (2020) found that complex upper limb movements can be classified by spatially selected electroencephalogram (EEG) data. Current techniques allow decoding different movements from EEG signals, but the accuracy of the results is still far from expected (Ofner et al., 2017). The H-reflex (the Hoffman reflex, a monosynaptic reflex of spinal cord) and V-wave (an electrophysiological index) are the most commonly used methods to measure changes in cortical excitability, but they can only show that neural adaptive resistance training is mediated at the “spinal” level and cannot determine the exact location (Schubert et al., 2008; McNeil et al., 2013). Several studies have used transcranial magnetic stimulation (TMS) to explore the response of the motor cortex to resistive motion, but this method has also proved to have contradictory results (Beck et al., 2007; Ansdell et al., 2020).

Functional near-infrared spectroscopy (fNIRS) is used to analyze the fine movement of the hand or finger, and there are few studies on the whole upper limb movement. fNIRS has been used to explore the degree of brain activity in patients with unilateral cerebral palsy during bilateral upper limb movement, and the results provided novel findings related to the control of bimanual tasks in unilateral cerebral palsy (de Campos et al., 2020). Complex hand movements activated different brain regions, and are different from simple movements (Li et al., 2020).

Functional near-infrared spectroscopy is a non-invasive brain imaging technique based on the principle of neurovascular coupling (Villringer and Chance, 1997; Leff et al., 2011). It detects changes caused by brain activity, disease, or injury by measuring changes in the concentration of oxygenated hemoglobin (HbO), deoxyhemoglobin, and total hemoglobin in the cerebral cortex (Zephaniah and Kim, 2014). fNIRS has the characteristics of device portability, low cost, easy data acquisition, and high temporal resolution compared with functional magnetic resonance imaging (fMRI), which can provide key information for the correct interpretation of brain responses related to motor performance (Perrey and Besson, 2018). fNIRS signals are less susceptible to movement than EEG signals (Perrey, 2008). Compared with fMRI, fNIRS can get quantitative and separate detection of HbO and deoxyhemoglobin levels by detecting the change of continuous near-infrared light, and can also be used to detect the oxygenation level of muscle tissue during exercise (Volkening et al., 2016; Pan et al., 2019).

EEG-based studies have already shown that motion execution, imagery, planning, and observation involved participation in both parietal and frontal lobes (Catrambone et al., 2019). In this study, we focused on the motor cortex and some cognitive regions, mainly including the parietal and frontal lobes. This study aims to explore the response of the cerebral motor cortex in different motor modes based on fNIRS. More specifically, fNIRS signals were collected to explore the difference in cortical activation area and degree when the upper limb moves along different trajectories. Moreover, resistance and non-resistance motion were tested to explore the brain response under the same trajectory in different modes.

## Materials and Methods

### Participants

Thirty healthy participants (12 females; 18 males) between the age of 22 and 25 (mean age 23.9 ± 0.9) participated in this study. All participants were right-handed as assessed by the Edinburgh Handedness scale. None had any motor or neurological impairments (self-reported), and all of them are non-smokers. Participants with the following conditions were excluded: (1) people with upper extremity motor dysfunction; (2) people with cardiovascular and cerebrovascular diseases; (3) people who stay up late before the experiment; and (4) people who consume alcohol, caffeine or drugs that may affect cardiovascular indicators within 8 h before the experiment. All participants provided written informed consent.

### Tasks Design and Apparatus

**FIGURE 1:**
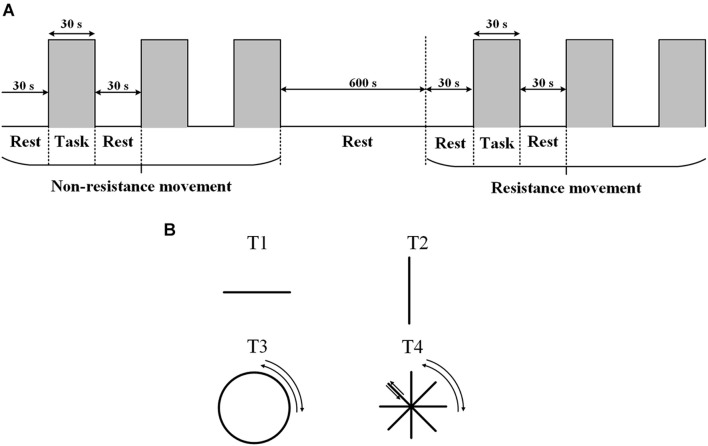
The task paradigm. **(A)** The experiment consists of two parts: non-resistance movement and resistance movement. Each task contains three periodic motions: rest for 30 s and exercise for 30 s. The interval between the two exercises is 600 s. **(B)** Four trajectories.

The experiment was carried out in a quiet room with a suitable temperature (about 25°C), dim light, and no electrical interference. Each participant was familiar with the experimental process before the experiment. During the experiment, the participants sat on a chair and faced a screen. The participants held the upper limb exercise training handle with the right hand, and the forearm was fixed to the handle. The table was adjusted to the appropriate height (about the same level as the heart). The experiment started with 30 s of rest with eyes closed, followed by 30 s of active non-resistance trajectory training. Each group of movements was carried out three times, with a total of four different trajectories (T1: left and right straight-line movement; T2: front and back straight-line movement; T3: clockwise and anticlockwise drawing circle movement; and T4: clockwise and anticlockwise character ⁕ movement). Four kinds of trajectories were selected, and the specific trajectories are shown in Figure 1B. During each 30 s trajectory movement, the participants moved back and forth along the same track at an appropriate speed (average number of movements were T1:15 times; T2: 15 times; T3: 3 times; and T4: 15 times). During the non-resistance movements, participants were able to move the handle smoothly with no resistance. Resistance training was set with omnidirectional resistance, and the resistance value was about 6 N. At the end of the non-resistance movement, the participants rested for 600 s. During the resting period, the optodes were not removed. The participant then carried out the same trajectory training with resistance (Figure 1). The upper limb rehabilitation training system (ArmGuider, ZD Medtech, China) was used to complete the trajectory training.

### Data Recording

**FIGURE 2:**
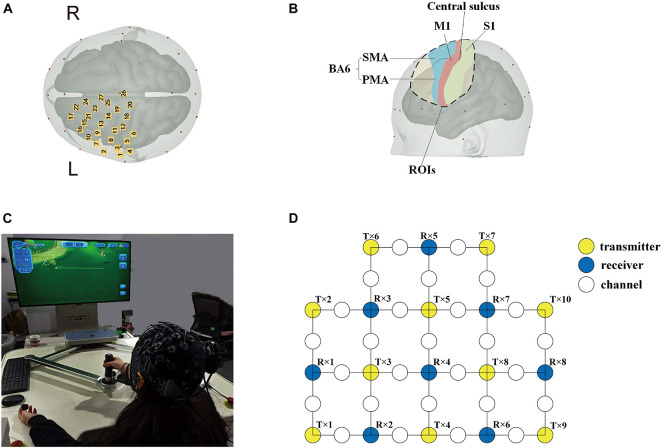
Basic experimental setup. **(A)** Cerebral cortical channel placement; **(B)** a map of the regions of the brain covered by light poles. Blue: supplementary motor area (SMA) and pre-motor area (PMA); green: the primary somatosensory cortex (S1); and red: primary motor cortex (M1). **(C)** Experimental environment and process diagram; **(D)** schematic diagram of 27 channels. Yellow circles: 10 transmitters; blue circles: 8 receivers; and white circles: 27 channels.

In this study, an fNIRS brain imager (Brite24, Artinis, Netherlands) was used to collect continuous cerebral cortex hemoglobin concentration waves. The device is equipped with 8 transmitters and 10 receivers, which constitute 27 channels in total. The principle of this method is to continuously and non-invasively detect the hemoglobin level of the cerebral cortex through the transmitter and receiver that emit infrared light with constant frequency and amplitude. According to the principle of contralateral control of the brain, these channels were set mainly to cover the supplementary motor area (SMA) and pre-motor area (PMA) in the frontal lobe Brodmann Area (BA) (BA6, BA8), the primary motor cortex (M1) in the frontal lobe (BA4), the primary somatosensory cortex (S1) in the parietal lobe (BA1, 2, 3), and the dorsolateral prefrontal area (DLPFC) in prefrontal lobe (BA9). The location of the optodes was marked with a 3D digitizer (FASTRAK, Polhemus, Colchester, VT, United States). These regions of interests (ROIs) have been shown to be associated with the activation of the cerebral cortex involved in active hand movement (Lee et al., 2018; Sagari et al., 2020; Rasooli et al., 2021), the anatomic labeling are listed in Table 1. The sampling frequency of the equipment is 10 Hz, and the distance between transmitter and receiver is 3 cm. The specific experimental setup is shown in Figure 2.

**TABLE 1:** Anatomic labeling of functional near-infrared spectroscopy (fNIRS) channel position, Brodmann areas (BAs).

Near-Infrared Spectroscopy (NIRS) channel	Brodmann areas	Description (ROI)
1, 4, 5	1, 2, 3	Primary somatosensory cortex
2, 7, 8, 9, 11, 12, 13, 14, 18, 19, 23, 25, 27	6, 8	Supplementary motor and pre-motor area
3, 6, 20, 26	4	Primary motor cortex
10, 15, 16, 17, 21, 22, 24	9	Dorsolateral prefrontal area

### Data Processing

The software package NIRS_SPM, based on MATLAB, was used to analyze the experimental data about HbO (Chul et al., 2009). The changes of light, the physiological state of participants, and the instability of instruments will affect the accuracy of the signal. To improve the signal-to-noise ratio, the hemodynamic response function (HRF) and the discrete cosine transform (DCT) were used. Principal component analysis (PCA) was employed to remove the unknown global trends due to breathing, cardiac, vaso-motion, or other experimental errors (Santosa et al., 2020). The general linear analysis model (GLM) was used to analyze the changes of HbO concentration (ΔHbO) in the cerebral cortex during the experiment.

The GLM was used to detect the active regions in the brain during the fNIRS analysis (Chen et al., 2020). The value of β reflects the activation degree of each channel, and the linear regression can be formulated as:

y=x⁢β+ε

where y represents ΔHbO and x is the predicted stimulation-evoked responses generated by convolving the task onset with the canonical HRF. β is the estimated amplitudes of ΔHbO, and ε is the error term (Zheng et al., 2021).

A cut-off frequency of 0.004 Hz high-pass filter was used to remove the baseline drift caused by the temperature change of the equipment during tasks. A correlation-based signal improvement (CBSI) method was utilized to correct motion artifacts (Cui et al., 2010).

### Statistical Analysis

The Shapiro-Wilk test was used to verify the normality of the data. The Pearson correlation coefficient was used for functional connectivity analysis (FC). In this study, vales of ΔHbO were extracted for subsequent analysis, which proved its superiority in evaluating functional activity (Zhang et al., 2017). The activation diagram of each experimental condition was obtained by a *t*-test after the group analysis conducted on the experimental data, and the Lipschitz-Killing curvature (LKC) based expected Euler characteristics (EC) correction. The ΔHbO of each subject under each trajectory was calculated, and a 27 × 27 symmetric correlation matrix was obtained by measuring the Pearson correlation coefficient of each channel time series. To obtain the group average FC, the individual FC of each subject in each trajectory was averaged, and then a 27 × 27 matrix of four group levels was generated. The two-way ANOVA was used to detect the significance of the activation between the four trajectories. The Bonferroni correction was used to correct for multiple comparisons. Furthermore, the time series of oxyhemoglobin changes during the task were generated to show the dynamic response of the cerebral cortex during exercise.

## Results

**FIGURE 3:**
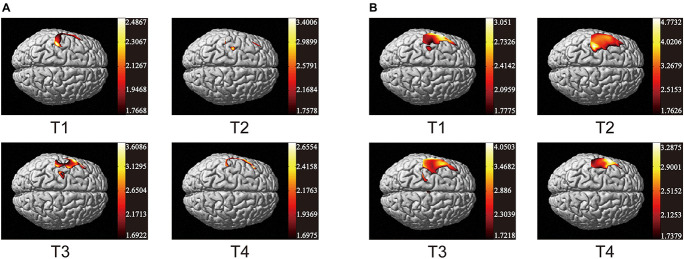
Activation maps of cerebral cortex from trajectory 1 to trajectory 4. **(A)** Non-resistance movement; **(B)** resistance movement. The change from red to yellow indicates that the degree of activation is from low to high. The coordinates in the figure show the activation range of the cerebral cortex in each mode. The data are *t* values, t:statistical value of sample *t*-test [with a significance level of *p* < 0.05, Lipschitz-Killing curvature (LKC)-based expected Euler characteristics (EC) correction]. The data and maps are calculated and generated by SPM_NIRS.

As the data in Figure 3 shows, both non-resistance exercises and resistance exercises activated the cerebral cortex. For non-resistance exercises, the different activation of the cerebral cortex is observed when the participants move along four different trajectories. T2 and T4 had no significant effect on cerebral cortex activation. T2 only activated part of the prefrontal cortex (CH7 and CH10) and S1 (CH5 and CH6), while T4 only activated parietal and frontal areas (CH1–CH6). The parietal lobe and frontal lobe were significantly activated by T1 (CH1–CH7 and CH18), and the activation of S1, M1, SMA, and PMA in T3 was also obvious (CH2–CH8, CH11, and CH12). For resistance exercises, the activation range significantly increased. CH1–CH8 were generally activated, and T2 was the most significant one for CH6. CH11 and CH12 were also activated. Generally, the response of the cerebral cortex to resistance movement was stronger than that of non-resistance movement.

**FIGURE 4:**
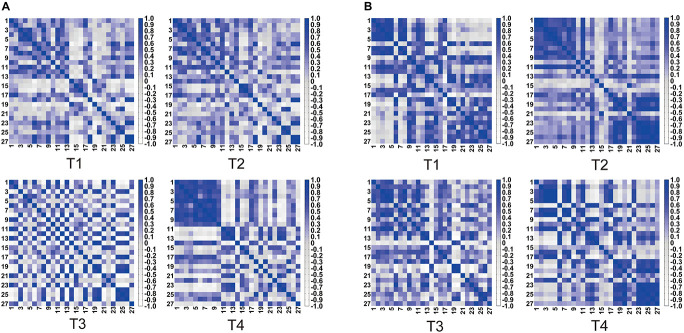
Symmetric heat maps of T1, T2, T3, and T4, which represent the Pearson correlation index of 27 channels. **(A)** Non-resistance movement; **(B)** resistance movement. The change from white to dark blue indicates that the correlation level is from negative 0.8 to positive 1.

Figure 4 shows the heat maps of four kinds of trajectory motion matrix during non-resistance exercises and resistance exercises separately. Each pixel value in the 27 × 27 matrix corresponds to a value of Pearson correlation coefficient, which represents the correlation between two measurement channels. It can be concluded that the correlations between the channels under four trajectories were quite different. The correlation of each channel was evenly distributed among T1, T2, and T3 under non-resistance movement. CH1–CH10 had a relatively strong correlation under T4. The correlation generally increased under resistance movement.

**FIGURE 5:**
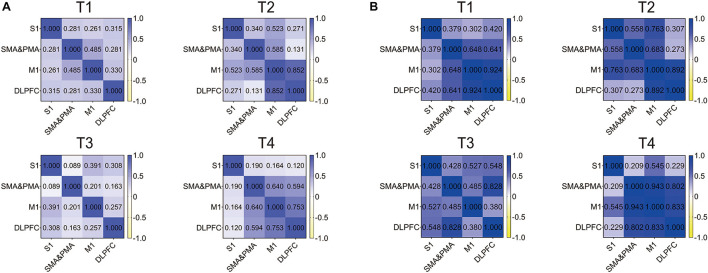
Correlation heat map of regions of interests (ROIs) in T1, T2, T3, and T4 for the primary somatosensory cortex (S1), supplementary motor area (SMA), pre-motor area (PMA), primary motor cortex (M1), and dorsolateral prefrontal cortex (DLPFC). **(A)** Non-resistance movement; **(B)** resistance movement. The color bar from yellow to dark blue indicates the correlation from –1 to 1.

We detected correlations between the ROIs in four kinds of trajectories for resistance movement and non-resistance movement. As observed in Figure 5, the correlation of all regions was not strong under T1 and T3 during non-resistance movement, and the overall correlation was <0.5. For T4, the correlation between each pair of regions was over 0.5 except S1. M1 and DLPFC had the strongest correlation during non-resistance movement under T2–T4, and the values were more than 0.75. Compared with non-resistance movement, the correlations were increased slightly during resistance movement.

**FIGURE 6:**
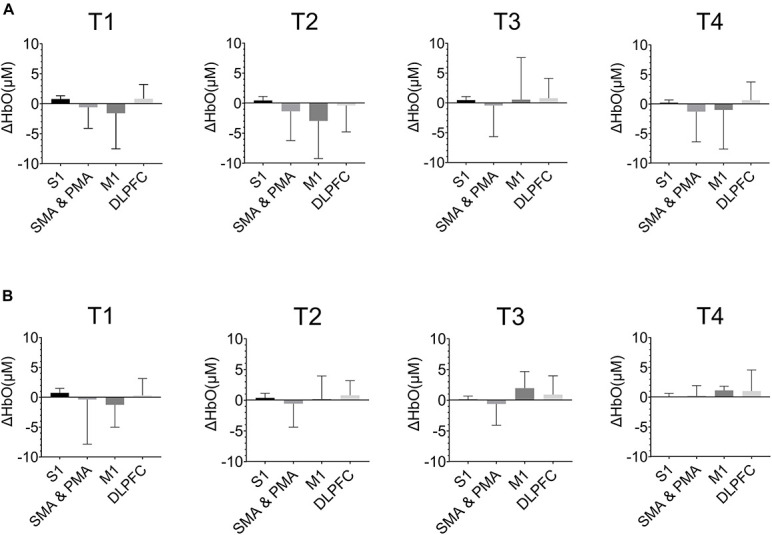
The HbO concentration (ΔHbO) in the cerebral cortex of regions of interests (ROIs) in T1, T2, T3, and T4. **(A)** Non-resistance movement; **(B)** resistance movement. Values were shown with mean + SD.

Figure 6 showed the ΔHbO in the cerebral cortex of ROIs in T1, T2, T3, and T4 for non-resistance movement and resistance movement. A *t*-test was used to detect the differences between each pair of regions, with a significance level of *p* < 0.05. For T1, activation during non-resistance movement and resistance movement was slightly different. For T2, there was significant activation of SMA, PMA, and M1, and this activation was enhanced in the resistance movement. Activation of DLPFC was slightly enhanced in non-resistance movement. For T3, the activation of M1 was observed to be enhanced during resistance movement. For T4, S1 had almost no activation but SMA, PMA, and M1 had a significant difference. There were significant differences between each pair of regions under both resistance movement and non-resistance movement (*p* < 0.0001).

**FIGURE 7:**
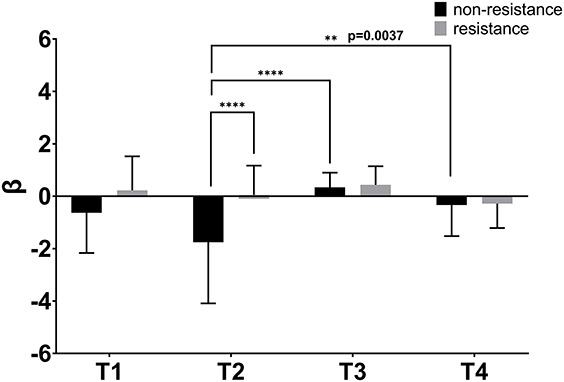
β values of four trajectories under two movement modes. The abscissa shows four trajectories. The coordinate is the value of β. β was extracted from the general linear analysis model (GLM). The bar indicates the errors. ^∗∗^*p* < 0.01, ^∗∗∗∗^*p* < 0.0001.

The two-way ANOVA was used to detect the significance of the activation between the four trajectories. As can be seen in Figure 7, the difference between the four trajectories was generally more significant under non-resistance movement compared with resistance movement. There were significant differences between T2 and T3 in non-resistance movement (*p* < 0.0001). Significant differences were observed between T2 and T4 in non-resistance movement (*p* = 0.0027). Only T2 had a statistical significance between non-resistance movement and resistance movement (*p* < 0.0001). The *post hoc* test showed that there was an interaction between two factors [*F*_(__3,196__)_ = 6.239, *p* = 0.0005].

**FIGURE 8:**
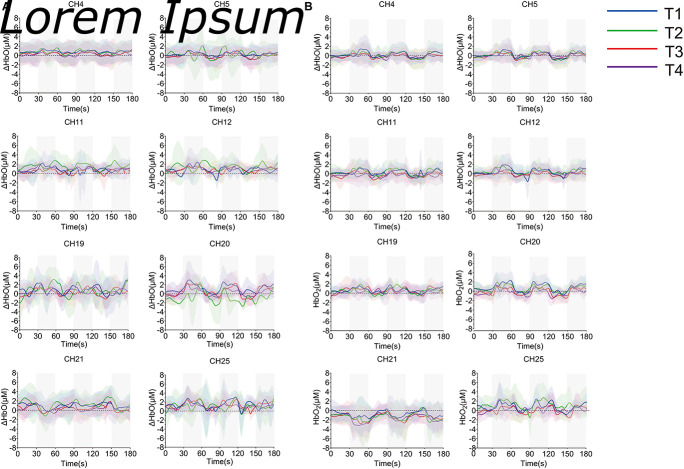
Time series of oxyhemoglobin changes during the task. **(A)** Non-resistance movement; **(B)** resistance movement. The gray area is the stimulus period. The solid line in the figure represents the average value of concentration, and the upper and lower shaded parts represent the error (mean ± SD) (*n* = 24).

The averaged time series of all tasks were shown in Figure 8. Due to the abnormal values of CH19, CH20, and CH21, six participants were eliminated from the group analysis. According to the degree of response, eight channels from S1 (CH4, CH5), SMA and PMA (CH11, CH12, CH19, and CH25), M1 (CH20), and DLPFC (CH21) were selected. There were no significant differences among the motions during non-resistance movement. The task state and the resting state were distinguished more obviously in the resistance movement. T2 had significant changes in CH20 during non-resistance movement, but there was no obvious periodicity in the changes of T2. The responses of CH19 and CH25 were irregular during resistance movement.

## Discussion

This research investigated the different responses of the cerebral cortex to different upper limb movement modes. The subjects performed upper limb exercise along four different trajectories in both resistance mode and non-resistance modes. Changes in oxyhemoglobin concentration in the cerebral cortex were recorded by fNIRS equipment.

Previous studies showed that active hand movement was associated with activation of SMA, M1 and S1 (Lee et al., 2018). In the present study, the motor cortex was activated, and the activated degrees were very different in two modes. Specifically, the brain activation degree of resistance exercise is significantly higher than that of non-resistance exercise. In Figure 3, the PMA and SMA regions are more active in the resistance movement, which is consistent with the results from the correlation heat map of each channel. The correlation between channels (1, 2, 3, 7, 8, 10, 11, and 12) becomes stronger, which indicates that regions covered by these channels participate in the regulation process of movement together. This change may be related to more intense stimulation to corresponding muscles and the increase of subjective participation during resistance exercise. The correlation between M1 and DLPFC was significant in both resistance and non-resistance movements. M1 was mainly responsible for planning, controlling, and executing movements, especially for any actions related to the delayed response (Chang et al., 2015). DLPFC was involved in higher-order cognitive functions and information processing related to attention and inhibition of responses (Karunakaran et al., 2021). Therefore, strong connectivity between M1 and DLPFC may be due to the need for more attention and participation in the task itself. It was found that movement-related cortical potentials (MRCP) changed during resistance exercise (Falvo et al., 2010). Most TMS experiments have observed that motor cortical inhibition decreased with resistance training (Goodwill et al., 2012; Weier et al., 2012; Leung et al., 2017), and it was consistent with the results of this study.

It is known that different regions of the brain have different regulatory functions on motor processes, and that different motor patterns cause different responses in the brain. As Figure 6 has shown, certain inverse oxygenation was observed. This may be due to the task mode and task complexity (Holper et al., 2011). The higher the complexity, the higher the probability of inverse oxygenation generally. However, T1 and T2 actually had lower complexity, and the inverse oxygenation may be due to unexpected artifacts. In our study, inverse oxygenation mainly occurred in SMA, PMA, and M1, and this was similar to previous studies (Mihara et al., 2012; Kempny et al., 2016). Considering the poor spatial resolution of fNIRS, adjacent areas may cause out-of-phase activation and this could be misinterpreted as inverse oxygenation (Abdalmalak et al., 2020).

In this study, the difficulty of the four trajectories is different, and the activation of the brain is different in short-term exercise. Among them, the activation of the brain under T3 was relatively high and involved more regional regulation. Compared with T1 and T2, T3 and T4 were associated with curve motion and had high complexity of the motion and difficulty of tracking. Different responses of the cerebral cortex may also be due to the inevitable physical movement of the subjects during the exercise. The elbow, shoulder, and most muscles of the upper limbs are involved in T3 and T4, and the range of motion was wide. Moreover, the motion involved in the experiment was active motion, therefore it is difficult to maintain absolute consistency between different subjects (Ubeda et al., 2015). Generally, different motion patterns are distinguished by motion planning, decision-making, and execution (Mohseni et al., 2020). The subjects needed to invest more energy and more rigorous control of the handle to follow the circular trajectory, i.e., T3 and T4, which was considered as the main reason for the greater response of the cerebral cortex.

In the compensation-related utilization of the neural circuits hypothesis (CRUNCH), healthy adults will employ more brain regions to participate in activities with an increased task load (especially PFC) (Reuter-Lorenz and Cappell, 2008; Herold et al., 2019). The positive changes in executive performance and brain activation are associated with cognitive performance improvements (Kujach et al., 2018; Wu et al., 2018). This shows that physical intervention had an important role in maintaining brain health and cognitive function. Different training can stimulate different muscle fibers, and the activation of the cerebral cortex is also different. As the load increases, the difference increases.

For subsequent studies, it is necessary to distinguish different motor patterns from electromyogram (EMG) signals and explain the brain’s regulation of different motor patterns from the perspective of muscle nerves. A multimodal approach based on fMRI techniques may be needed to better understand neural plasticity in cortical networks in terms of spatial refinement and multidimensional numbers. Moreover, the actual upper limb movement should not be limited to the two-dimensional plane, and later research may need to extend the motion trajectory to the three-dimensional space. Furthermore, it is necessary to recruit patients who need exercise rehabilitation (such as stroke patients) to provide a clinical reference. Considering that the difference of motion itself involved in this study is not obvious, later studies may need to consider more complex planned motion and introduce EMG and other methods to explore the mechanisms of muscle-neurovascular coupling.

## Conclusion

This study explored the different responses of the cerebral motor cortex in different upper limb motor patterns. The response of the cerebral cortex to four trajectories was different, and the activation under clockwise and anticlockwise drawing circle movement was the most obvious. Meanwhile, we found a more significant activation during resistance movement, and the participation of the SMA and PMA regions was higher. This study provides reference value for evaluating the efficacy of upper limb motor rehabilitation.

## Data Availability Statement

The datasets presented in this article are not readily available because data may include privacy of participants. Requests to access the datasets should be directed to PS, rehabishi@163.com.

## Ethics Statement

Ethical review and approval was not required for the study on human participants in accordance with the local legislation and institutional requirements. The patients/participants provided their written informed consent to participate in this study.

## Author Contributions

PS and AL performed the research and data analysis. HY helped with the concept, methodology development, and interpretation of data. PS and AL drafted the manuscript. All authors approved the manuscript.

## Conflict of Interest

The authors declare that the research was conducted in the absence of any commercial or financial relationships that could be construed as a potential conflict of interest.

## Publisher’s Note

All claims expressed in this article are solely those of the authors and do not necessarily represent those of their affiliated organizations, or those of the publisher, the editors and the reviewers. Any product that may be evaluated in this article, or claim that may be made by its manufacturer, is not guaranteed or endorsed by the publisher.
